# Clinical Characteristics and Nomogram for Predicting Mortality in Patients with Postoperative Bloodstream Infection in Surgical Intensive Care Unit

**DOI:** 10.1155/2024/9911996

**Published:** 2024-01-12

**Authors:** Zengli Xiao, Yao Sun, Huiying Zhao, Youzhong An

**Affiliations:** The Department of Critical Care Unit, Peking University People's Hospital, Beijing 100044, China

## Abstract

**Background:**

Bloodstream infection is amongst the leading causes of mortality for critical postoperative patients. However, data, especially from developing countries, are scary. Clinical decision-making tools for predicting postoperative bloodstream infection-related mortality are important but still lacking.

**Objective:**

To analyze the distribution of pathogens and develop a nomogram for predicting mortality in patients with postoperative bloodstream infection in the surgical intensive care unit.

**Methods:**

The clinical data, infection and pathogen-related data, and prognosis of patients with PBSI in the SICU from January 2017 to January 2022 were retrospectively collected. The distribution of pathogens and clinical characteristics of patients with PBSI were analyzed. The patients were assigned to a died group and a survived group according to their survival status. Independent predictors for mortality were identified by univariate and multivariate analyses. A nomogram for predicting PBSI-related death was developed based on these independent predictors. Calibration and decision-curve analysis were established to evaluate the nomogram. We collected postoperative patients admitted to our center from February 2022 to June 2023 as external validation sets to verify the nomogram. We also add the Brier score to further validate the model.

**Results:**

In the training set, 7128 patients admitted to the SICU after different types of surgery were collected. A total of 198 patients and 308 pathogens were finally enrolled. The mean age of patients with PBSI was 64.38 ± 16.22 (range 18–90) years, and 56.1% were male. Forty-five patients (22.7%) died in the hospital. Five independent predictors including BMI, APACHE II score, estimated glomerular filtration rate (eGFR), urine volume in the first 24 hours after surgery, and peak temperature before positive blood cultures were selected to establish the nomogram. The area under the receiver operating characteristic curve for the prediction model was 0.922. Calibration curve and decision curve analysis showed good performance of the nomogram. Seventy patients with PBSI were collected as an external validation set, and thirteen patients died in this set. The external validation set was used to validate the nomogram, and the results showed that the AUC was 0.930 which was higher than that in the training set indicating that the nomogram had a good discrimination. The brier score was 0.087 for training set and 0.050 for validation set.

**Conclusions:**

PBSI was one of the key issues that clinicians were concerned and could be assessed with a good predictive model using simple clinical factors.

## 1. Introduction

Bloodstream infection (BSI) is defined by positive blood cultures in patients with systemic signs of infection. Hospital-acquired BSIs are one of the most common and serious complications in postoperative patients, especially those admitted to the intensive care unit (ICU) [1, 2]. Postoperative bloodstream infections (PBSIs) are associated with prolonged ICU and hospital stays, higher costs, and also increased rates of morbidity and mortality [3–6]. The incidence of BSIs and the types of pathogens may vary depending on the different types of surgery. But most previous studies have focused on a certain specific type of surgical procedure, especially intraabdominal and cardiac surgery [7, 8]. BSI data following all kinds of surgery was rare.

To evaluate the pathogen characteristics of PBSIs following different types of surgeries and risk factors for mortality in patients with PBSI, we analyzed the recent five-year data in the surgical intensive care unit (SICU) of our hospital, which is a tertiary care hospital located in the capital area of China. In addition, early identification of high-risk patients using clinical and laboratory examinations is of major importance for a promising therapy, followed by immediately initiated procedures for source control and an adequate anti-infective therapy. But risk factors and a simplified clinical decision-making tool for identifying these high-risk patients are still lacking. Nomograms are widely used as prognostic devices in medicine [9]. With the ability to generate an individual probability of a clinical event by integrating diverse prognostic and determinant variables, nomograms meet our desire for clinically integrated models and fulfill our drive towards personalised medicine. So we try to develop a nomogram based on these independent predictors for predicting PBSI-related death.

## 2. Methods

### 2.1. Definitions

BSI was postoperative if the patient had been operated on during the same hospital stay or within 30 days of the BSI, or if BSI was secondary to the surgical site infection [10]. Diagnosis of BSI caused by a potential skin contaminant required a temperature >38.5°C or <36°C, chills, hypotension (systolic blood pressure less than 90 mmHg), and antimicrobial therapy temporally associated with the positive blood culture result.

### 2.2. Study Cohort

We conducted a retrospective study at Peking University People's Hospital, China. All patients who were operated in the operating room and admitted to the SICU from January 2017 to January 2022 were evaluated as the training set. Patients admitted into SICU from February 2022 to June 2023 were collected as a validation set. All patients with PBSI during the study period were reviewed. Cases with an incomplete record and an age <18 years old were excluded. Patients were assigned to *s* survived group and a died group according to their surviving status at the time of discharge from the hospital.

### 2.3. Data Collection

**Figure 1:**
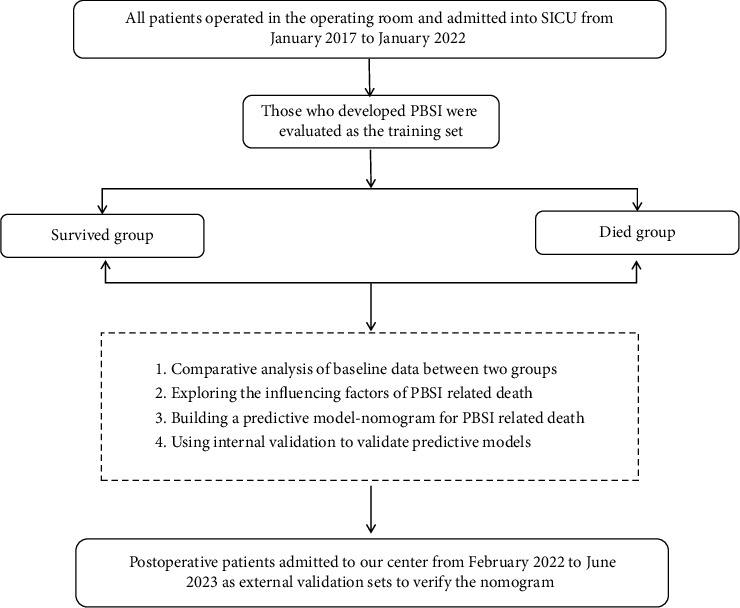
BSIs following different types of surgery.

Demographic characteristics, dates of hospital and SICU admission and discharge, admitting diagnosis, surgical procedures, underlying medical conditions, temperature, and infection-related laboratory indicators were recorded. Acute Physiology and Chronic Health Evaluation (APACHE II) scores were calculated on all patients during the first 24 hours of admission to the SICU. The manuscript workflow diagram is shown in Figure 1.

### 2.4. Data Analysis

Continuous variables with a normal distribution are presented as the mean and standard deviation (SD) and were analyzed using Student's *t*-test. Continuous variables with a nonnormal distribution are reported as median and interquartile range (IQR), and comparisons between groups were based on the Mann–Whitney *U* test. Categorical variables are summarized as numbers with percentages, and group comparisons were based on Fisher's exact test or chi-squared test. Univariate and multivariate logistic regression were performed to identify independent risk factors, and the nomogram was established based on independent risk factors. To qualify the predictive ability of the nomogram, the area under the curve (AUC) and the receiver operating characteristic curve (ROC) were used to evaluate the discrimination. The calibration curve was used to evaluate the calibration of the nomogram, and the decision curve analysis (DCA) was used to estimate the clinical usefulness of the nomogram by calculating the net benefits at different threshold probabilities. We also add the Brier score to further validate the model. Statistical analyses were performed using R statistical software. A *p* value <0.05 (two-sided) was considered significant in this study.

## 3. Results

### 3.1. BSIs following Different Types of Surgery

Between January 2017 and January 2022, 7128 patients were admitted to the SICU after different types of surgery. A total of 270 patients with positive blood cultures and 380 pathogens were identified. Seventy-one patients were excluded for suspected skin contamination and one patient was excluded for being less than 18 years old. One hundred and ninety-eight patients and 308 pathogens were finally enrolled in this study. The mean age of patients with PBSI was 64.38 ± 16.22 (range 18–90 years), and 56.1% were male. The mean duration of ICU and hospital stays for all patients was 24.87 ± 29.45 days and 43.76 ± 40.33 days. The highest burden of PBSI was observed after gastrointestinal surgery (151 isolates), followed by orthopaedic surgery (40), nervous system (30), and cardiovascular surgery (29). The rates were highest in the nervous system (23/349, 6.6%), followed by gastrointestinal surgery (3.5%). The rates were lowest in gynaecologic (1.5%) and cardiovascular surgery (1.7%) (Table 1).

**Figure 2:**
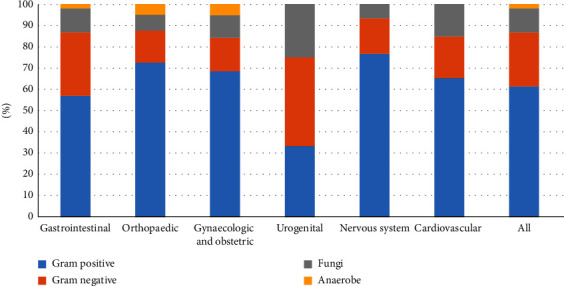
Nomogram for predicting PBSI-related mortality.

The causative agents of the BSIs varied by type of surgery (Figure 2). *Klebsiella pneumoniae*, *Acinetobacter baumannii*, and *Pseudomonas aeruginosa* were the top three common Gram-negative pathogens. *Coagulase-negative staphylococcus* and *Enterococcus faecium* were the most common Gram-positive pathogens. The most common pathogenic fungus was *Candida albicans* (Table 2).

### 3.2. Risk Factors for Mortality

Table 3 provides a comparison of survived and died patients. Forty-five patients (22.7%) died in the hospital. Patients in the died group had a significantly older age (70.7 ± 12.4 vs. 62.5 ± 16.8, *p*=0.003) and a lower BMI level (22.62 ± 4.75 vs. 24.97 ± 4.45, *p*=0.002) than that in the survived group. The most common comorbidities were hypertension (51%), followed by solid tumor (36.9%), diabetes (23.7%), and coronary atherosclerotic heart disease (23.7%) with no difference between the two groups. However, patients with end-stage renal dysfunction (ESRD) in the died group were significantly more than those in in the survived group (24.4% vs. 5.9%). The heart rate at the time of admission to the SICU (101.53 ± 25.2 vs. 92.23 ± 24.74, *p*=0.028) and the highest temperature before positive blood culture (39.36 ± 0.75°C vs. 38.83 ± 0.64, *p* < 0.001) were higher in the died group than those in the survived group. Compared with survived patients, patients who died had a significantly lower eGFR (47.06 ± 27.59 vs. 85.57 ± 34.44, *p* < 0.001) and less urine output in the first 24 hours after surgery (1519.13 ± 941.91 vs. 2019.57 ± 968.32, *p*=0.002). In addition, patients who died in hospital also had a higher APACHE II score than survived patients (20.33 ± 5.25 vs. 15.84 ± 2.85, *p* < 0.001).

Table 4 shows variables significantly associated with in-hospital mortality. They include BMI, APACHE II score, eGFR, urine volume in the first 24 hours after surgery, and peak temperature before positive blood cultures.

### 3.3. Nomogram for Predicting PBSI-Related Mortality

**Figure 3:**
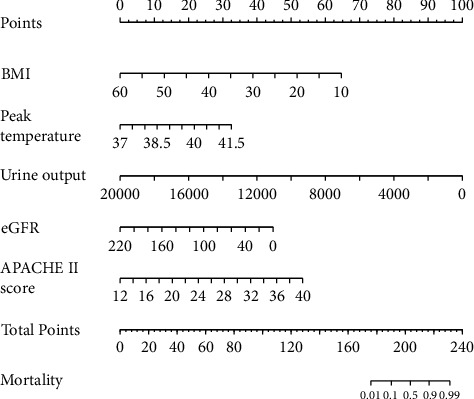
Internal calibration of the nomogram for predicting PBSI-related mortality.

**Figure 4:**
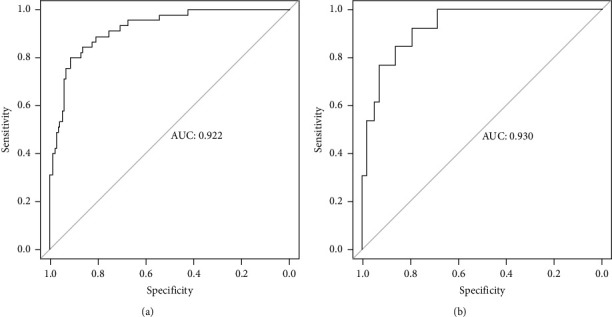
The AUC for the prediction nomogram model.

**Figure 5:**
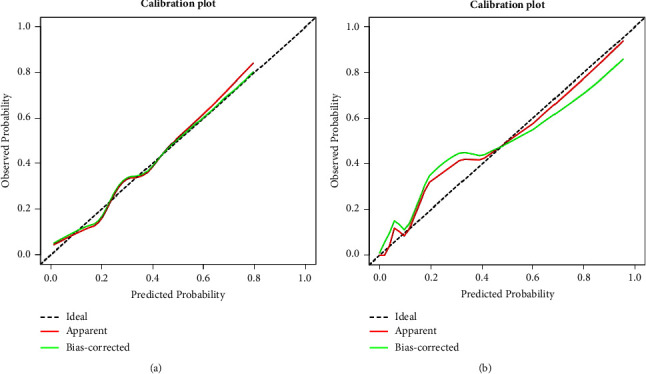
The decision curve of the nomogram.

**Figure 6:**
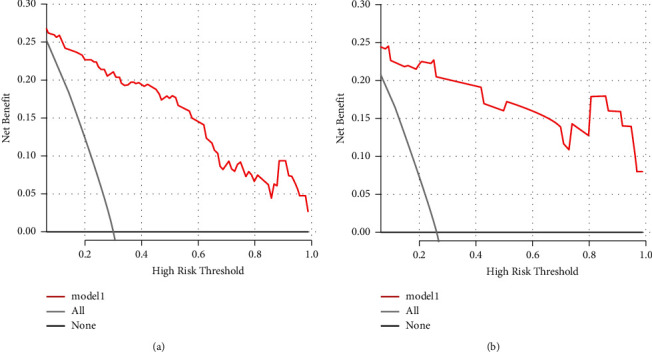
The external validation.

The independent predictors determined in this study were selected to establish the nomogram (Figure 3). The area under the receiver operating characteristic (ROC) curve (AUC) for the prediction model was 0.922, which showed a good accuracy in predicting adverse events of the TLE procedure (Figure 4(a)). The favorable calibration plot of our nomogram indicated that the prediction by the nomogram was highly consistent with the actual observation (Figure 5(a)). The clinical decision curve analysis is shown in Figure 6(a). The Brier score was 0.087 for the training set.

### 3.4. External Validation for the Nomogram

Two thousand three hundred and seventy-three postoperative patients were admitted to our center from February 2022 to June 2023 and seventy patients who developed PBSI were collected as external validation sets to verify the nomogram. The external validation set showed that the AUC was 0.930 which (Figure 4(b)) was higher than that in the training set indicating that the nomogram had a good discrimination. The clinical calibration curve and decision curve analysis of the validation set are shown in Figures 5(b) and 6(b). The brier score was 0.050 for the validation set.

## 4. Discussion

The present study is based on a fifteen-bed SICU in a tertiary care hospital including all specialities of surgery in China. Approximately 1500 postoperative patients were admitted to our SICU each year. To our knowledge, this study is the first one to evaluate the pathogen distributions and risk factors for mortality in critical patients with BSI following all kinds of surgeries in developing countries. We also developed the first nomogram for predicting mortality in patients with PBSI.

Nearly 2.8% (198/7128) patients presenting to the SICU during the study period fulfilled the PBSI criteria. It is difficult to compare PBSI data with other studies due to variation in definitions used to identify PBSI cases and the different patient populations in the SICU or general ward. Dr. Christophe Adrie analyzed 10734 ICU patients and identified 571(5.7%) patients who developed one or more ICU-acquired BSI [11]. However, 74.8% patients were from medical wards in their study. Skogberg et al. conducted a study including 427518 surgical procedures, and the overall rate of postoperative BSI was 1.7/1000 surgical procedures [10]. O'Brien et al. analyzed 659486 patients undergoing major surgery, and 23815 (3.6%) had a 30-day infection. Among these 23815 patients, 1906 (8%) had a bloodstream infection. This study proved that patients with a 30-day postoperative infection had a 3.2-fold higher risk of 1-year infection and a 1.9-fold higher risk of 1-year mortality [6]. Different from our study, the patients included in this study were not limited to those admitted to the ICU, and the postoperative infections included surgical site infection, pneumonia, urinary tract infection, and bloodstream infection.

The PBSI rate reported in our study was significantly higher than Skogberg's and O'Brien's studies. The reasons are as follows: first of all, most of the patients admitted to our hospital were from lower-level hospitals and complicated with a variety of underlying diseases. Second, patients admitted to our SICU are always those who had an extensive and complex operation or underwent a large intraoperative bleeding or circulatory fluctuations.

The most common causative pathogens were Gram-positive pathogens in this study which was higher than previous studies. This is because that most previous studies excluded all coagulase-negative *Staphylococcus* pathogens [2]. Since parts of PBSIs ensued from catheter-related infections including central venous catheter, urinary catheter, and various drainage tubes, which could result in coagulase-negative *Staphylococcus* pathogen infections, we did not exclude all coagulase-negative *Staphylococcus* pathogens.

We used the Charlson score to compare the comorbidities between the two groups. This is a comorbidity score that provides a mortality risk determination based upon the patient's comorbidity risk determination based upon the patient comorbidity [12, 13]. In the surgical patients, it was used to determine the risk of developing a postoperative sepsis [14, 15]. In our study, this score differed between died and survived patients, but this was not confirmed in the multivariate analysis. We also used the APCHE II score to measure the severity of illness at admission to the ICU before the development of the PBSI and found that the APCHE II score was significantly higher in the died group than in survived group.

The mortality of patients with PBSI in this study was 22.7% (45/198) in the training set and 21.6% (13/70) in validation set. The independent predictors for mortality including the lower BMI, lower eGFR level, higher APACHE II score, less urine volume, and higher temperature. These five factors were used to create the nomogram for predicting mortality in patients with postoperative bloodstream infections in the surgical intensive care unit. The nomogram has good predictive ability and could be used by any clinicians.

### 4.1. Limitations

The data in this study were from a single-center experience which may lead to some bias. Our center is a highly experienced, high-volume tertiary center in China. The patients referred to our hospital often had complex comorbidities which may not encountered at other centers. Due to the low rate of PBSI, the number of cases included in this study is relatively small, and the nomogram may be overfitting. Further prospective, multicenter studies with a larger series of patients are needed to increase the reliability and practicability of this model.

## 5. Conclusions

The mortality of patients with PBSI in the SICU remains high. This study creates a nomogram for predicting PBSI-related mortality which demonstrated a good predictive ability. The nomogram could help clinicians assess the patients at high risk and make a better and more suitable treatment decision for these postoperative patients.

## Figures and Tables

**Table 1:** PBSIs after different types of surgeries.

Surgical procedure category	Surgical procedures, *N*	All BSIs				Duration of ICU stay (days)	Duration of hospital stay (days)
		Isolates, *N*	Patients, *N*	Age, yrs (mean ± SD)	Male, *N*		
Gastrointestinal	2489	151	86 (3.5%)	69.43 ± 13.40	51 (59.3%)	23.57 ± 32.15	49.97 ± 49.65
Orthopaedic	1030	40	25 (2.4%)	62.84 ± 19.16	14 (56%)	29.80 ± 32.04	36.24 ± 31.03
Cardiovascular	1210	29	20 (1.7%)	64.15 ± 10.63	12 (60%)	30.95 ± 29.91	46.30 ± 30.77
Peripheral vessels	340	17	11 (3.2%)	59.00 ± 20.41	7 (41.2%)	31.18 ± 33.42	53.64 ± 32.79
Chest wall, pleura, mediastinum, diaphragm, trachea, bronchus, and lungs	256	8	7 (2.7%)	68.14 ± 17.91	4 (57.1%)	18.57 ± 10.93	31.86 ± 10.87
Gynaecologic	807	15	12 (1.5%)	53.17 ± 14.85	0	18.45 ± 14.96	50.07 ± 26.12
Obstetric	115	4	3 (2.6%)	30.33 ± 4.99	0	24.50 ± 36.58	39.00 ± 12.19
Nervous system	349	30	23 (6.6%)	59.09 ± 19.16	14 (60.9%)	22.57 ± 23.94	25.17 ± 27.79
Urogenital	475	12	10 (2.1%)	68.3 ± 10.71	8 (80%)	23.40 ± 27.30	40.22 ± 31.10
Minor surgical procedures^*∗*^	57	2	1 (1.8%)	26.00 ± 0.00	1 (100%)	7 ± 0	18 ± 0
All	7128	308	198	64.38 ± 16.22	111	24.87 ± 29.45	43.76 ± 40.33

^∗^Surgical procedure categories with less than 10 patients admitted to SICU per year including teeth, jaws, mouth, and breast surgeries.

**Table 2:** Pathogen distribution.

Isolates	Number	Percentage (%)
Gram negative		
*Klebsiella pneumoniae*	19	6.17
*Acinetobacter baumannii*	12	3.90
*Pseudomonas aeruginosa*	11	3.575
*Stenotrophomonas maltophilia*	9	2.92
*Escherichia coli*	7	2.27
*Enterobacter cloacae*	8	2.60
*Serratia marcescens*	3	0.97
Others	9	2.92
Gram positive		
*Staphylococcus epidermidis*	63	20.45
*Staphylococcus hominis*	43	13.96
*Staphylococcus capitis*	19	6.17
*Enterococcus faecium*	17	5.52
*Staphylococcus haemolyticus*	15	4.87
*Staphylococcus aureus*	6	1.95
*Enterococcus faecalis*	7	2.27
Others	19	6.17
Fungi		
*Candida albicans*	17	5.52
*Candida parapsilosis*	6	1.95
*Candida glabrata*	6	1.95
Others	1	0.32
Anaerobe	6	1.95
Total	308	100

**Table 3:** Baseline characteristics of 198 patients diagnosed with PBSI in SICU.

	All patients (*n* = 198)	Mortality status (all causes)		*p*
		Survived (*n* = 153)	Died (*n* = 45)	
Age (years)	64.37 ± 16.26	62.5 ± 16.8	70.7 ± 12.4	**0.003**
**Males**	111 (56.1%)	82 (53.6%)	29 (64.4%)	0.233
BMI (kg/m^2^)	24.44 ± 4.62	24.97 ± 4.45	22.62 ± 4.75	**0.002**
Charlson comorbidity index	2.42 ± 1.83	2.24 ± 1.72	3.04 ± 2.07	**0.009**
CCI > 3	55 (27.6%)	34 (22.1%)	21 (27.6%)	**0.002**
Underlying conditions				
Diabetes	47 (23.7%)	35 (22.9%)	12 (26.7%)	0.69
Hypertension	101 (51.0%)	81 (52.9%)	20 (44.4%)	0.397
Coronary atherosclerotic heart disease	47 (23.7%)	33 (21.6%)	14 (31.1%)	0.231
History of myocardial infarction	11 (5.6%)	7 (4.6%)	4 (8.9%)	0.275
Arrhythmia	27 (13.6%)	19 (12.4%)	8 (17.8%)	0.336
Chronic liver disease	33 (16.7%)	23 (15.0%)	10 (22.2%)	0.261
Solid tumor	73 (36.9%)	56 (36.6%)	17 (37.8%)	1.000
End stage renal dysfunction	20 (10.1%)	9 (5.9%)	11 (24.4%)	**0.001**
Cerebrovascular disease	39 (19.7%)	26 (17.0%)	13 (28.9%)	0.09
Emergency procedure	54 (27.3%)	39 (25.5%)	15 (33.3%)	0.342
Time of operation	5.04 ± 2.48	4.93 ± 2.35	5.41 ± 2.89	0.256
Blood loss during operation	859.32 ± 1588.66	783.76 ± 1220.78	1116.22 ± 2462.56	0.218
Laboratory data				
C-reactive protein	134.27 ± 89.00	128.43 ± 86.12	154.45 ± 96.66	0.088
Procalcitonin	10.72 ± 22.80	10.26 ± 24.01	12.36 ± 18.00	0.603
Heart rate	94.34 ± 25.09	92.23 ± 24.74	101.53 ± 25.20	**0.028**
Peak temperature	38.96 ± 0.70	38.83 ± 0.64	39.36 ± 0.75	**0.001**
MAP, mean (SD)	106.51 ± 24.99	106 ± 24.57	107.82 ± 26.64	0.691
eGFR	76.81 ± 36.62	85.57 ± 34.33	47.06 ± 27.59	**0.001**
Urine output	1905.83 ± 982.77	2019.57 ± 968.32	1519.13 ± 941.91	**0.002**
No. of BSI episodes	1.56 ± 0.97	1.48 ± 0.84	1.84 ± 1.30	**0.026**
1	126 (63.6%)	102 (66.7%)	24 (53.3%)	1.000
2	50 (25.3%)	37 (24.2%)	13 (28.9%)	0.56
≥3	22 (11.1%)	14 (9.2%)	8 (17.8%)	0.112
APACH II score on ICU admission	16.86 ± 4.00	15.84 ± 2.85	20.33 ± 5.25	**0.001**
Outcome (days)				
ICU length of stay	24.87 ± 29.45	22.42 ± 22.81	33.20 ± 44.67	**0.031**
Hospital length of stay	43.76 ± 40.33	41.54 ± 32.85	51.31 ± 58.99	0.153

**Table 4:** Risk factors for mortality.

	B	Wald	OR	CI	*p*
BMI	−0.223	9.426	0.800	0.694–0.922	0.002
APACHE II score	0.327	18.75	1.387	1.196–1.609	0.001
eGFR	−0.031	12.198	0.969	0.952–0.986	0.001
Urine volume	−0.001	5.733	0.999	0.999–1.000	0.017
Peak temperature	1.416	12.173	4.122	1.860–9.135	0.001

B, coefficient estimates; Wald, chi-square value; OR, odds ratio; CI, confidence interval.

## Data Availability

The data that support the findings of this study are available on request from the corresponding author. The data are not publicly available due to privacy or ethical restrictions.
